# Phenolics and Carbohydrates in Buckwheat Honey Regulate the Human Intestinal Microbiota

**DOI:** 10.1155/2020/6432942

**Published:** 2020-02-26

**Authors:** Li Jiang, Minhao Xie, Guijie Chen, Jiangtao Qiao, Hongcheng Zhang, Xiaoxiong Zeng

**Affiliations:** ^1^Institute of Apicultural Research, Chinese Academy of Agricultural Sciences, Beijing 100093, China; ^2^College of Food Science, Nanjing Agricultural University, Nanjing 210095, China; ^3^National Research Center of Bee Product Processing, Ministry of Agriculture, Beijing 100093, China

## Abstract

Intestinal microbiota plays an important role in human health. The aim of this paper is to determine the impact of the phenolics and carbohydrate in buckwheat honey on human intestinal microbiota. We investigated the phenolics and carbohydrate compositions of eight buckwheat honey samples using high-performance liquid chromatography and ion chromatography. The human intestinal microbes were cultured in a medium supplemented with eight buckwheat honey samples or the same concentration of fructooligosaccharides. The bacterial 16S rDNA V4 region sequence of DNA extraction was determined by the Illumina MiSeq platform. 12 phenolics and 4 oligosaccharides were identified in almost all buckwheat honey samples, namely, protocatechuic acid, 4-hydroxy benzoic acid, vanillin, gallic acid, *p*-coumaric acid, benzoic acid, isoferulic acid, methyl syringate, *trans,trans*-abscisic acid, *cis,trans*-abscisic acid, ferulic acid, 4-hydroxybenzaldehyde, kestose, isomaltose, isomaltotriose, and panose. Most notably, this is the first study to reveal the presence of 4-hydroxybenzaldehyde in buckwheat honey. 4-Hydroxybenzaldehyde seems to be a land marker of buckwheat honey. Our results indicate that buckwheat honey can provide health benefits to the human gut by selectively supporting the growth of indigenous *Bifidobacteria* and restraining the pathogenic bacterium in the gut tract. We infer that buckwheat honey may be a type of natural intestinal-health products.

## 1. Introduction

Honey, as a crucial natural sweetener, has been used approximately for six thousand years and was the only origin of the sweeteners for a long time. Honey is collected by honeybees from the nectar, blossoms, or the secretions of living parts in plants [1]. Honeybees combine the nectar with their own specific substances and then deposit, dehydrate, and ripen it in honeycombs [2]. Generally, honey can be divided into two groups, multifloral honeys and monofloral honeys. The most common monofloral honeys include acacia honey, linden honey, longan honey, lychee honey, and buckwheat honey. Buckwheat honey is known as “black honey” with sweet, delicious taste, long aftertaste, and unique malt flavor. Buckwheat honey seems to be one of the most valued honeys due to health benefits [3]. The abilities of buckwheat honey to promote health may be significantly correlated with its phenolic and carbohydrate compounds.

Phenolics and carbohydrates are central compounds in buckwheat honey. Phenolics are defined as the secondary metabolic products of the plants [4]. Phenolics include flavonoids and phenolic acids, with more than 5000 compounds already described [5]. Previous work claimed that main phenolics in buckwheat honey were caffeic acid, protocatechuic acid, *p*-hydroxybenzoic acid, *p*-hydroxyphenylacetic acid, syringic acid, *p*-coumaric acid, ferulic acid, and *p*-hydroxybenzoic acid; moreover, *p*-coumaric acid and *p*-hydroxybenzoic acid were the essential components [6]. Another research reported that buckwheat honey contained protocatechuic acid, caffeic acid, ferulic acid, gallic acid, and *p*-coumaric acid. Gallic acid and *p*-coumaric acid were the primary constituents [7]. It is worth noting that these studies show obvious disagreement about the phenolic composition of buckwheat honey. In addition, honey is a supersaturated carbohydrate solution with more than 75% carbohydrates [8]. As is well known, honey carbohydrates are made up of about 70% monosaccharides (glucose and fructose) and 10% oligosaccharides. Honey oligosaccharides vary in constitution as well as degree of polymerization. Unfortunately, specific carbohydrate composition in buckwheat honey is unclear.

In recent years, intestinal microbes have become a central issue for human health. Nobel Prize winner Joshua Lederberg pointed out that the human body and body symbiotic microorganisms constitute a super organism. Recent evidence indicates that the role of gut microbes extends beyond the gut. Gut microbiota impacts several functions in systemic organs such as the liver [9] and brain [10]. In addition, intestinal microbes closely relate to immune, nutrition, and other physiological functions [11]. The number of microbes in the adult intestine reaches 10^14^, close to human body cells, and weight reaches 1.2 kg, close to that of the human liver. Genes in intestinal microbes are 100-fold more than those in human cells [12]. More studies have shown that host intestinal flora affects food digestion; moreover, various foods also impact the constituents of intestinal microbes. Some food ingredients are digested in the human small intestine, while the nondigestible compounds are metabolized by bacteria in large intestine. Various bacteria can metabolize food compounds to different substrates, which also affect the dominant intestinal microbes. Previous work claimed that intestinal microbes were regulated by phenolics and carbohydrates. According to Parkar SG, phenolics may confer health benefits for gut through affecting the total number of beneficial microbes in the gut [13]. Shin and Ustunol explored the effect of three honeys on intestinal bacteria, including sourwood honey, alfalfa honey, and sage honey. Their study stated that carbohydrates of honey provided health benefits by selectively supporting the growth of indigenous *Bifidobacteria* in the gastrointestinal microflora tract and reducing the gut pH as a result of the production of lactic and acetic acids [14]. Honey seems to inhibit the potentially deleterious bacteria from existing among the intestinal microflora. Therefore, honey can be used to overcome various gastrointestinal diseases and endow the beneficial management of gut microflora [15]. Ancient Chinese believed that buckwheat honey can improve gut health. For example, about 1800 years ago, the celebrated ancient doctor named Zhongjing Zhang transformed liquid honey into soft solid forms and then placed soft solid honey in human anus to cure constipation. Additionally, the famous Compendium of Materia Medica claimed that one of buckwheat honey's functions was to regulate the gastrointestinal system. However, there remains a need for providing reliable evidence as to how buckwheat honey modulates intestinal microorganisms.

**Figure 1:**
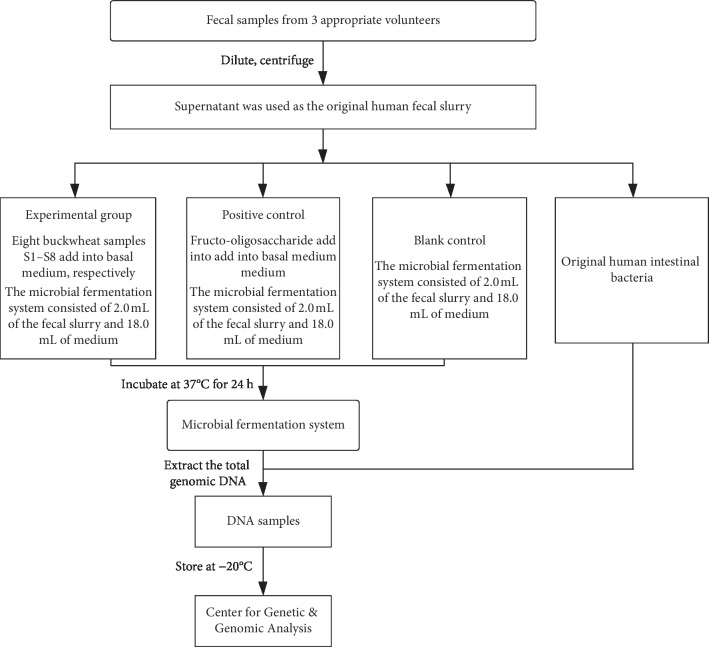
Flow diagram of anaerobic fermentation in vitro and DNA extraction.

The aim of the present paper is to determine the effect of the phenolics and carbohydrates in buckwheat honey on the growth and activity of intestinal microbes (Figure 1). Our results will gain a better insight as to how buckwheat honey may modify the gut microflora.

## 2. Materials and Methods

### 2.1. Reagents, Chemicals, and Materials

Standards of *cis,trans*-abscisic acid and *trans,trans*-abscisic acid were obtained from Chengdu Biotechnology Company (Chengdu, China); standards of benzoic acid, *p*-coumaric acid, ferulic acid, isoferulic acid, gallic acid, methyl syringate, vanillin, 4-hydroxybenzaldehyde, 4-hydroxybenzoic acid, fructose, and glucose were obtained from Sigma-Aldrich Chemical Company (St. Louis, MO, USA); standards of kestose, isomaltose, isomaltotriose, and panose were obtained from Aladdin Company (Shanghai, China); sodium hydroxide solution was obtained from Sigma-Aldrich Chemical Company (St. Louis, MO, USA); fructooligosaccharides were obtained from Shanghai Yuanye Biotechnology Company (Shanghai, China).

Methanol (analytical grade and HPLC grade) was obtained from Thermo Fisher Scientific Inc. (Fair Lawn, NJ, USA); formic acid (analytical grade) and acetic acid (HPLC grade) were purchased from J. T. Baker Inc. (Phillipsburg, NJ, USA); ultrapure water was purified using a Milli-Q-Integral System (Millipore, Billerica, MA, USA); the Strata-X-A (60 mg/3 mL) cartridge was obtained from Phenomenex Inc. (Torrance, CA, USA); a 24-port VisiprepTM solid-phase extraction vacuum was obtained from Sigma-Aldrich-Supelco (St. Louis, MO, USA) and was used for all preconcentration procedures. Cysteine, 0.85% stroke-physiological saline solution, agar, NaCl, K_2_HPO_4_, MgSO_4_, CaCl_2_, NaHCO_3_, and Tween 80 were obtained from Sigma-Aldrich Chemical Company; peptone, yeast extract, and chlorhematin were obtained from Solarbio Company (Beijing, China); bile salt, resazurin, and vitamin K1 were obtained from Yuanye Biotechnology Company; sealed boxes, anaerobic gas agents, and oxygen indicator paper were obtained from Mitsubishi Chemical Corporation (Tokyo, Japan).

Heraeus Biofuge Stratos high-speed benchtop centrifuges and ICS-3000 ion chromatography (Dionex) were from Thermo Fisher Company (Fair Lawn, NJ, USA). The purification column is Dionex OnGuard II RP Cartridge, 2.5-cc, Pkg. of 48; DNA extraction kit was from Tiangen Biochemical Technology Company (Beijing, China); LS-50HD vertical steam sterilizer was obtained from Jiangyin Binjiang Medical Equipment Company (Jiangsu, China); Thermo Scientific Heracell 150i incubator is from Thermo Fisher Scientific Inc.

### 2.2. Composition of Buckwheat Honey

#### 2.2.1. Honey Sample Preparation

Eight buckwheat honey samples were collected from 8 different areas of China. These apiaries were located in Inner Mongolia (97°12′–126°04′E, 37°24′–53°23′N, sample 1), Heilongjiang (121°11′–135°05′E, 43°26′–53°33′N, samples 2 and 3), Liaoning province (118°53′–125°46′E, 38°43′–43°26′ N, sample 4), Jilin province (121°38′–131°19′E, 40°50′–46°19′N, sample 5), Henan province (31°23′–36°22 ′E, 110°21′–116°39′N, sample 6), Shanxi province (105°29′–111°15′E, 31°42′–39°35′N, sample 7), and Beijing (115°42′–117°24′E, 39°24′–41°36′N, sample 8). All honey samples were stored at 4°C prior to analysis.

#### 2.2.2. Phenolic Compounds and Abscisic Acid of Buckwheat Honey Samples

Phenolics were extracted using solid-phase extraction (SPE) based on the method of our previous study [16]. To determine phenolics and abscisic acid concentrations in honey samples, we used an HPLC system with a PDA-20A diode array detector, SIL auto-injection valve, CTO-10A thermostat, and LC-6AD pump (Shimadzu Corporation, Tokyo, Japan), equipped with a quadrupole time-of-flight mass spectrometer (Q-TOF MS) (Agilent 6540, Agilent Technologies, Palo Alto, CA, USA). A reversed-phase Gemini C18 column (150 × 4.6 mm, 5 *μ*m) (Phenomenex Inc., Torrance, CA, USA) was used. The mobile phase consisted of 2% acetic acid in water (phase A) and 2% acetic acid in methanol (phase B). The flow rate was 0.7 mL/min, and the temperature of the column oven was set at 35°C. A 100-minute linear gradient was performed as follows: 0–11 min, 3–8% B; 11–14 min, 8–10% B; 14–17 min, 10–14% B; 17–24 min, 14–20% B; 24–28 min, 20–21% B; 28–30 min, 21-22% B; 30–38 min, 22–25% B; 38–41 min, 25–30% B; 41–46 min, 30–33% B; 46–55 min, 33% B; 55–60 min, 33-34% B; 60–70 min, 34–36% B; 70–75 min, 36–52% B; 75–85 min, 52–57% B; 85–95 min, 57–65% B; 95–100 min, 65–80% B.

Phenolic compounds and abscisic acid were authenticated by comparison of their retention times and UV spectra with those of standards and identified by HPLC-Q-TOF-MS. MS was operated with 4 kV source voltage, 130 V capillary voltage, and 350°C capillary temperature. All MS data were acquired in the positive and negative ionization modes. The quantification of 11 phenolic compounds and abscisic acid was based on the peak area using external calibration curves at a wavelength of 280 nm.

#### 2.2.3. Carbohydrates of Buckwheat Honey

Preprocessing: (1) about 0.2 g of uniformly mixed buckwheat honey samples was transferred into a 100 mL volumetric flask, fully dissolved in 20 mL of warm water, and cooled to room temperature; then water was added to volume; (2) the sample solutions were shaken to ensure that they completely dissolved; (3) purification column was activated with 10 mL methanol and 15 mL water and put after half an hour into use; (4) the sample solutions were passed through 0.45 *μ*m filter and purification column in sequence, the previous 3 column volumes of eluent were discarded, and the subsequent eluent was collected to be tested on the machine.

Carbohydrates were analyzed by ICS-3000 ion chromatography (Dionex) with a CarboPac™ PA1 column (4 ∗ 250 mm). The mobile phase consisted of ultrapure water (phase A) and 200 mmol/L NaOH solution (phase B). The flow rate was 1 mL/min, and the temperature of the column oven was set at 30°C. A 15-minute process was performed on ion chromatography.

### 2.3. Intestinal Microbiota Detection

#### 2.3.1. Cultures Preparation

The fecal samples were obtained from three volunteers (two males and one female, 22–28 years old) who did not have any gastrointestinal disease or take antibiotics, prebiotics, probiotics, laxatives, or narcotics over the past 3 months. The three fecal samples were mixed together and then mixed with sterilized saline (containing 0.5 g/L cysteine). The mixture was diluted into 10% fecal suspension, and then the fecal suspension was centrifuged with 300 ×g at 4°C for 5 minutes. The supernatant was regarded as the original human intestinal bacteria.

The composition of culture medium per liter was as follows: 2.0 g yeast extract, 0.1 g NaCl, 0.04 g K_2_HPO_4_, 0.04 g KH_2_PO_4_, 0.01 g MgSO_4_, 0.01 g CaCl_2_, 2.0 g NaHCO_3_, 0.02 g chlorhematin, 0.5 g cysteine, 0.5 g cholate, 1.0 mg resazurin, 2.0 mL Tween 80, and 10 *μ*L vitamin K1.

#### 2.3.2. Growth Determination and Sequencing

Eight buckwheat honey samples from different geographical sources were added to the culture medium at a concentration of 10 g/L. The same concentration of fructooligosaccharides was used as a positive control. Culture medium without the addition of fructooligosaccharides and buckwheat honey were regarded as a blank control. Bacterial suspension of 2 mL was mixed with 18 mL mediums, and then the mixture was put into triangular bottles. Triangular bottles were placed in sealed boxes. Anaerobic gas agents were added into the sealed boxes to remove oxygen, and then oxygen indicator paper was added to ensure the oxygen partial pressure was less than 0.1%. Anaerobic culture systems were cultured in static culture at 37°C and manually oscillated every six hours to make system average as possible. After 24 hours, the microorganism was obtained by centrifugation from the culture medium, and microbial DNA was extracted from the fecal DNA extraction kit.

The high-throughput sequencing of the bacterial 16S rDNA V4 region was performed on an Illumina MiSeq platform by Genesky Biotechnologies Inc. (Shanghai, China). The operational taxonomic units (OTUs) were clustered and annotated on the basis of the Ribosomal Database Project (RDP) database by UPARSE with 97% similarity cutoff [17, 18]. Refraction curves and alpha diversity (Shannon and Simpson indexes) were calculated with Mothur [19]. Beta diversity analyses, including principal component analysis (PCA) and nonmetric multidimensional scaling (NMDS), were performed using R software (Version 2.15.3) and vegan package. The contributions of carbohydrate and polyphenols in buckwheat honey to the microbial profile were analyzed by redundancy analysis (RDA).

The data are expressed as the mean ± standard error mean. The least significant difference (LSD), Duncan's multiple range test, and one-way analysis of variance (ANOVA) were used for multiple comparisons by SPSS 22. *P* < 0.05 was considered to be of statistical significance.

## 3. Results

### 3.1. Phenolic Compounds and Abscisic Acids in Buckwheat Honey Samples

**Figure 2:**
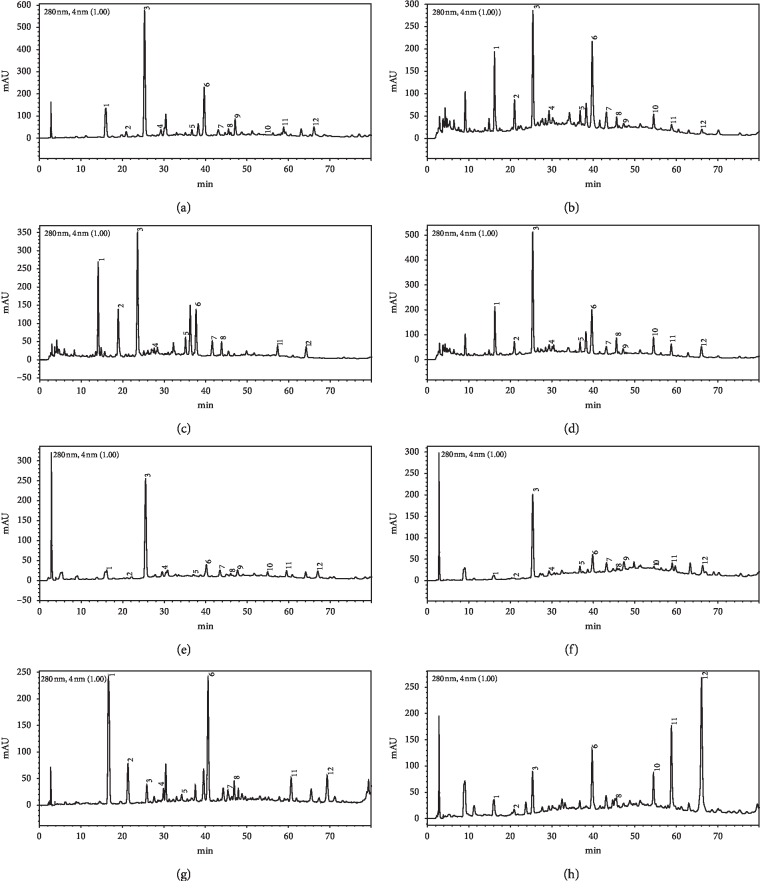
HPLC profile of buckwheat honey samples. Note. (1) protocatechuic acid, (2) 4-hydroxy benzoic acid, (3) 4-hydroxybenzaldehyde, (4) vanillin, (5) gallic acid, (6) *p*-coumaric acid, (7) benzoic acid, (8) ferulic acid, (9) isoferulic acid, (10) methyl syringate, (11) *trans,trans*-abscisic acid,(12) *cis,trans*-abscisic acid. (a) Sample 1, (b) sample 2, (c) sample 3, (d) sample 4, (e) sample 5, (f) sample 6, (g) sample 7, (h) sample 8.

HPLC profiles of buckwheat honey samples were extremely complex with many peaks between the retention time of 10 and 70 min (Figure 2). According to the HPLC profiles of phenolic extracts and UV spectra's major peaks, we identified 12 compounds, including 10 phenolic acids and esters, *cis,trans*-abscisic acid, and *trans,trans*-abscisic acid. It is worth noting that peak 2, peak 3, and peak 7 were identified as 4-hydroxy benzoic acid, 4-hydroxybenzaldehyde, and benzoic acid, respectively. Samples 1, 2, 3, and 4 exhibited very similar chromatograms; peak 3 is the highest. In addition, peak 3 was also the highest in samples 5 and 6. Thus, 4-hydroxybenzaldehyde seems to be a land marker of buckwheat honey.

Table 1 shows the contents of phenolic and abscisic acid in eight buckwheat honey samples. As can be seen, the highest content of 4-hydroxybenzaldehyde was found in all samples except samples 7 and 8. The content ranged from 55 *μ*g/100 g to 1092 *μ*g/100 g honey with an average content of 567.32 *μ*g/100 g. 4-Hydroxybenzaldehyde represented nearly 40% of total phytochemicals in samples 1 to 6. The highest content of 4-hydroxybenzoic acid was observed in sample 2 with the value of 275 *μ*g/100 g honey, and the lowest was in sample 5 with 43 *μ*g/100 g honey.

### 3.2. Carbohydrates in Buckwheat Honey Samples

Honey is mainly composed of 80% carbohydrates [20].  Table 2 illustrates major carbohydrates in buckwheat honey. As can be seen, six kinds of carbohydrate were distinguished in all the honey samples, namely, fructose, glucose, kestose, isomaltose, isomaltotriose, and panose. Fructose showed the highest level ranging from 40.4% to 48.7%. The second high content was glucose ranging from 21.6% to 28.7%. Kestose, isomaltose, isomaltotriose, and panose presented a small amount in all buckwheat honey samples.

### 3.3. Diversity of Intestinal Microbiota in Samples

Experimental groups (S1–S8), positive control (FOS), blank control (BLK), and the human original microbes (ORI) were used to assess the effects of buckwheat honey on intestinal microorganisms. We performed a high-throughput sequencing on the Illumina MiSeq platform. Our results revealed a total of 1278684 reads. They had passed all quality filters under 97% identity conditions to obtain a total of 5954 species classification OTUs. On average, there were 180 OTUs for each sample. High-coverage Illumina data showed that the sequence specificity reached 99.9%. The alpha diversity was estimated based on the observed Chao 1, Shannon, and Simpson indexes and reflected the community diversity of single samples (Table 3). The observed Chao 1 reflected the richness of species within a single sample, while Shannon and Simpson indexes represent microbial diversity [21]. Shannon indexes were positively correlated with diversity; in comparison, Simpson was negatively correlated with diversity. As seen in Table 3, the Chao 1 and Shannon indexes of buckwheat honey (S1–S8) and FOS were lower than those of blank control (*P* < 0.05), and the Simpson of buckwheat honey (S1–S8) and FOS were higher than those of blank control (*P* < 0.05). Thus, this indicates that the buckwheat honey (S1–S8) and FOS exhibited lower diversity.

**Figure 3:**
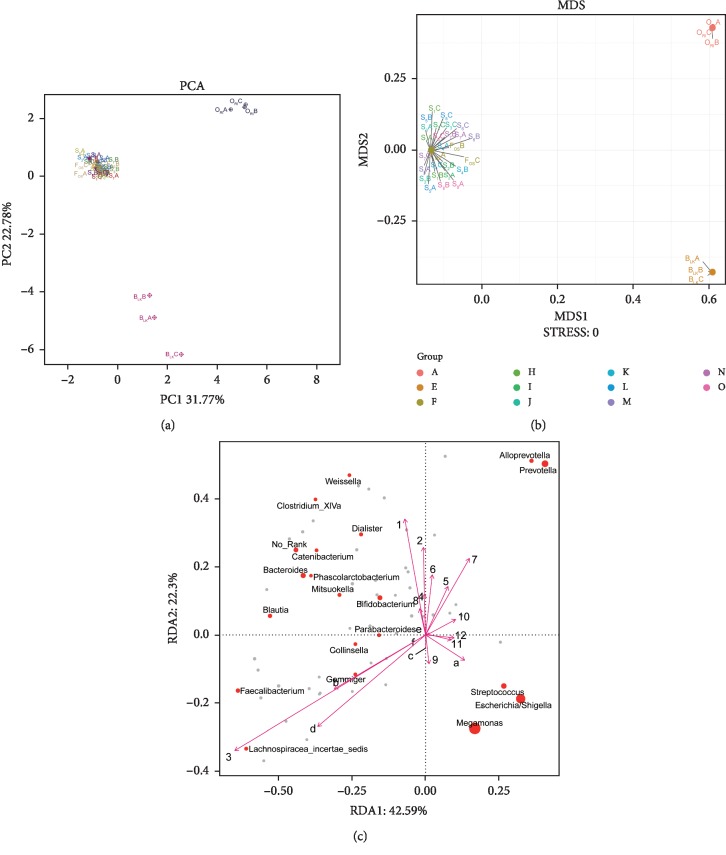
The PCA (a), NMDS (b), and RDA (c) analysis. Note: In (a) and (b), S1A, S1B, and S1C represent the fermentation triplicate of buckwheat honey sample S1. FOSA, FOSB, and FOSC represent the fermentation triplicate of positive control fructooligosaccharides. BLKA, BLKB, and BLKC represent the fermentation triplicate of blank control. ORIA, ORIB, and ORIC represent the fermentation triplicate of the human original microbes. (c): 1. protocatechuic acid; 2. 4-hydroxy benzoic acid; 3. 4-hydroxybenzaldehyde; 4. vanilin; 5. gallic acid; 6. *p*-coumaric acid; 7. benzoic acid; 8. ferulic acid; 9. isoferulic acid; 10. methyl syringate; 11. *trans*,*trans*-abscisic acid; 12. *cis*,*trans*-abscisic acid. a. fructose; b. glucose; c. kestose; d. isomaltose; e. isomaltotriose; f. panose.

Figure 3 presents the PCA (A), NMDS (B), and RDA (C) analysis of experimental group, positive control, blank control, and original human intestinal microbes. S_1_A, S_1_B, and S_1_C represent the fermentation triplicate of buckwheat honey sample S1. F_OS_A, F_OS_B, and F_OS_C represent the fermentation triplicate of positive control fructooligosaccharides. B_LK_A, B_LK_B, and B_LK_C represent the fermentation triplicate of blank control. O_RI_A, O_RI_B, and O_RI_C represent the fermentation triplicate of the human original microbes. Intestinal microorganism was visualized on a two-dimensional structure to illustrate clustering, based on the phylogenetic distance. As can be seen, PCA and NMDS analysis of experimental group and positive control cluster overlap, suggesting the similar effect of buckwheat honey and fructooligosaccharides on the human intestinal microflora. However, blank control was separated completely from the experimental group and positive control, suggesting the significant difference. The contributions of polyphenols and carbohydrates in buckwheat honey to the microbial composition were determined by RDA (Figure 3(c)). In RDA, the first and second ordination axes were plotted, explaining 42.59% and 22.3% of the variance. Polyphenols and carbohydrates were separated significantly in RDA analysis (*P* < 0.05). Polyphenols dominated the modulation on intestinal microbiota, suggesting obvious impact on the fermentation in vitro on both RDA1 and RDA2 compared to carbohydrates.

**Figure 4:**
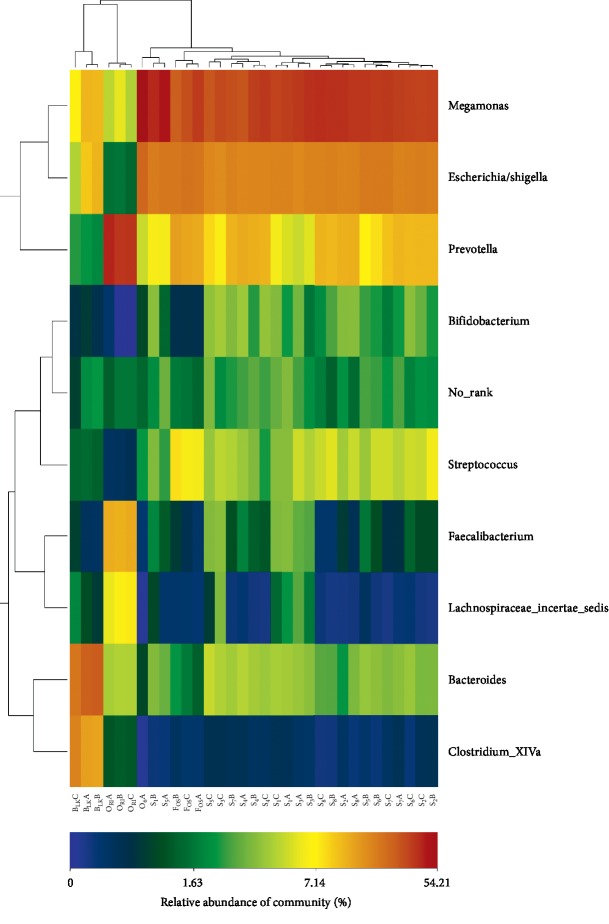
The heatmap of major gut microbiota relative abundance in genus level. Note. S_1_A, S_1_B, and S_1_C represent the fermentation triplicate of buckwheat honey sample S1. FOSA, FOSB, and FOSC represent the fermentation triplicate of positive control fructooligosaccharides. B_LK_A, B_LK_B, and B_LK_C represent the fermentation triplicate of blank control. O_RI_A, O_RI_B, and O_RI_C represent the fermentation triplicate of the human original microbes.

Gut microbiota are recognized as having a significant effect on health. The microbiota have both digestive and metabolic functions [22] and play an essential role in the development of the host immune system [23]. Extensive evidence has shown that core microbes are responsible for maintaining human healthy state [24, 25].  Figure 4 reveals the heatmap of major gut microorganism relative abundance from experimental group, positive control, blank control, and original human intestinal microbes. At the genus level, a total of 9 bacteria were identified. We can find that the abundance of intestinal microbes in experimental group, positive control, blank control, and original human intestinal microbes was different. In comparison with the blank control, *Megamonas* in the experimental group and positive control was increased (*P* < 0.05). In addition, the abundance of *Escherichia/Shigella*, *Bifidobacterium*, and *Streptococcus* also increased (*P* < 0.05). In contrast, *Prevotella*, *Faecalibacterium*, and *Lachnospiraceae incertae sedis* decreased (*P* < 0.05). It is worth noting that the *Bifidobacteriaceae* level of the experimental group was higher than that of positive control (*P* < 0.05).

## 4. Discussion

Prior studies have noted that intestinal microbes are essential to human health. The contributions of intestinal microorganism to nutrition are multifaceted [26]. For example, intestinal microbes can promote synthesis and assimilation of vitamins and macronutrients, aid fat absorption, and regulate host glucose and energy metabolism. In this study, we tested the effect of buckwheat honey on human intestinal microbiota based on Illumina MiSeq platform.

Phenolics are vital substances contributing to sensory and quality properties, such as color, taste, or flavor of honey [27]. Our results indicate that buckwheat honey contains an extensive number of phenolics. Twelve phenolics were identified in almost all buckwheat honey samples, namely, protocatechuic acid, 4-hydroxy benzoic acid, vanillin, gallic acid, *p*-coumaric acid, benzoic acid, isoferulic acid, methyl syringate, *trans,trans*-abscisic acid, *cis,trans*-abscisic acid, ferulic acid, and 4-hydroxybenzaldehyde (Table 1 and Figure 2). Four characteristic compounds exist in all samples and exhibit higher levels, that is, protocatechuic acid, 4-hydroxy benzoic acid, 4-hydroxybenzaldehyde, and *p*-coumaric acid. Compared with recent studies [6, 28], this is the first study to elucidate 4-hydroxybenzaldehyde in buckwheat honey. Among all samples, 4-hydroxybenzaldehyde possessed the highest content with 55 to 1092 *μ*g/100 g honey being more three times that of other compounds (Table 1). 4-Hydroxybenzaldehyde seems to be a land marker of buckwheat honey. It is worth noting that 4-hydroxybenzaldehyde, 4-hydroxy benzoic acid, and benzoic acid are well known for their antimicrobial activity. Prior works have demonstrated that honey possesses antimicrobial activity; for example, van den Berg reported that buckwheat honey exhibited significant inhibitory activity against *S. aureus* and *E. coli* [29]. Our results (Figure 4) are also consistent with the research of Lin et al. showing that honey may restrain the pathogenic intestinal microbes [30]. Thus, we speculate that 4-hydroxybenzaldehyde, 4-hydroxy benzoic acid, and benzoic acid in buckwheat honey can inhibit precarious microorganisms.

Our results show that buckwheat honey can regulate human intestinal microorganism. Buckwheat honey is particularly effective in enhancing the growth of probiotics. For example, in comparison with the abundance of *Bifidobacterium* in the blank control, the experimental group increased the abundance of *Bifidobacterium* (Figure 4 and Table 3). The growth of probiotics may be associated with carbohydrate in buckwheat honey. The principal carbohydrate constituents of buckwheat honey were fructose, glucose, and some oligosaccharides, including kestose, isomaltose, isomaltotriose, and panose (Table 2). The oligosaccharides in buckwheat honey may serve as probiotic agents to promote the growth of intestinal probiotics. Our findings are similar to the previous conclusion that the growth of intestinal *Bifidobacteria* was enhanced, probably due to the honey oligosaccharides with low degree of polymerization [31]. Mono- and disaccharides of honey are absorbed rapidly in the upper gut tract, so nondigestible oligosaccharides reach the lower gut tract to selectively impact colonic microflora. In addition, our results show that polyphenols also markedly affect intestinal microbiota (Figure 3(c)). Many studies have reported that dietary polyphenols may promote the proliferation of *Bifidobacterium*, such as green tea, persimmon polyphenols, and anthocyanins [32–35]. Our findings are also in agreement with Shin and Ustunol's finding that honey may support the growth of indigenous *Bifidobacteria* and inhibit growth of pernicious bacteria. However, the study of Shin and Ustunol did not determine entire human gut microorganism; they only chose to determine some intestinal microbes [14]. Our study focused on the effect of buckwheat honey on whole human intestinal microbes. Most notably, buckwheat honeys may have a relatively stronger effect on human intestinal microorganisms than fructooligosaccharides (Figure 4). Buckwheat honey is the comprehensive solution containing various carbohydrates and polyphenols. A previous study focuses on the impact of carbohydrates in honey on intestinal microbes, while few studies analyze the contributions of other dietary components, including polyphenols. Furthermore, Sanz et al. have reported that the prebiotic activity of honey is lower than fructooligosaccharides [36]. However, from the perspective of our present data, buckwheat honey is particularly effective in enhancing the growth of probiotics compared to the same concentration of fructooligosaccharides (Figure 4). Moreover, the carbohydrates exerted much less influence than polyphenols on microbiota (Figure 3(c)). We speculate that phenolics and oligosaccharides in buckwheat honey seem to synergistically impact human intestinal microbes. We assume that the synergic functions of multiple phenolics and oligosaccharides in buckwheat honey are more efficient than that of a single component. In addition, our results demonstrate that the main constituents of phenolic and carbohydrate were almost similar in eight samples from different geographical sources, and there were no significant differences in regulating intestinal microbes, including diversity and relative abundance. We speculate that the same monofloral honey from different geographical sources seems to play a similar role in regulating human intestinal microorganism. Overall, our study suggests that buckwheat honey may be salutary to human gut health. Our research was limited to the in vitro effects; thus, in the following study, we will focus on buckwheat honey regulating the human intestinal microbiota in vivo.

## 5. Conclusions

Our research shows that the characteristic compounds of buckwheat honey include 4-hydroxybenzaldehyde, *p*-coumaric acid, protocatechuic acid, 4-hydroxybenzoic acid, *cis,trans*-abscisic acid, and *trans,trans*-abscisic acid. To our knowledge, this is the first report that 4-hydroxybenzaldehyde is identified and seems to be a land marker in buckwheat honey. Four oligosaccharides exist in all buckwheat honey samples with a small amount, including kestose, isomaltose, isomaltotriose, and panose. Phenolics and oligosaccharides in buckwheat honey seem to synergistically impact human intestinal microbes to enhance the growth of probiotics. Furthermore, polyphenols dominated the modulation on intestinal microbiota. Our results seem to provide reliable evidence that buckwheat honey may regulate gut microflora. A future in vivo study still needs to be verified by human trials.

## Figures and Tables

**Table 1:** The contents of phenolics in buckwheat honey samples from different geographical origins (*μ*g/100 g honey).

Samples (*μ*g/100 g honey)	S1	S2	S3	S4	S5	S6	S7	S8
Benzoic acid	82.64	98.06	77.16	147.52	68.62	89.73	45.32	—
*cis,trans*-Abscisic acid	111.08	64.23	66.02	81.41	40.44	44.42	64.95	489.31
Gallic acid	109.55	182.15	83.65	150.52	63.47	66.17	38.25	—
Isoferulic acid	152.64	—	6.91	35.97	40.56	88.27	—	—
Methyl syringate	30.66	—	93.74	138.52	2.89	68.12	—	237.52
*p*-Coumaric acid	316.01	221.86	180.42	303.35	54.99	76.71	423.52	265.95
Ferulic acid	53.25	72.11	73.69	76.35	0.48	23.81	74.4	45.67
Protocatechuic acid	265.47	462.96	352.64	379.52	84.24	75.27	451.52	139.41
*trans,trans*-Abscisic acid	66.69	70.42	58.52	61.51	28.77	39.92	65.01	402.41
Vanillin	41.76	24.96	63.52	107.35	29.86	53.96	46.15	—
4-Hydroxybenzaldehyde	1092.35	564.04	1081.73	734.41	464.33	322.52	55.81	223.41
4-Hydroxy benzoic acid	65.79	275.43	188.89	231.21	43.22	48.58	72.96	64.25

Note. Average calculated from two independent analyses. (—): not detected. Sample 1 from Mongolia, sample 2 from Heilongjiang, sample 3 from Heilongjiang, sample 4 from Liaoning, sample 5 from Jilin, sample 6 from Henan, sample 7 from Shanxi, and sample 8 from Beijing.

**Table 2:** The contents of carbohydrates in buckwheat honey.

Samples (g/100 g honey)	S1	S2	S3	S4	S5	S6	S7	S8
Fructose	40.4	44.8	48.4	42.8	46.6	48.7	49.4	48.5
Glucose	28.4	24.4	25.1	23.6	28.7	21.6	22	24.8
Kestose	0.06	0.07	0.09	0.07	0.1	0.09	0.09	0.08
Isomaltose	1.4	0.41	0.64	0.47	0.54	0.49	0.52	0.57
Isomaltotriose	0.1	0.1	0.1	0.1	0.1	0.1	0.1	0.1
Panose	0.1	0.1	0.1	0.1	0.1	0.1	0.1	0.1

Note. Average calculated from two independent analyses.

**Table 3:** The reads, OTU, Ace, Chao 1, and coverage index at 0.03 level.

	Reads	0.03				
		OTU	Coverage	Chao 1	Shannon	Simpson
S1	38748	189	0.999088	223.3 ± 13.5	2.27 ± 0.20	0.246 ± 0.033
S2	38748	171	0.999028	215.0 ± 13.1	2.17 ± 0.07	0.256 ± 0.031
S3	38748	191	0.999036	230.0 ± 3.0	2.33 ± 0.14	0.234 ± 0.028
S4	38748	180	0.999071	216.0 ± 19.2	2.33 ± 0.12	0.222 ± 0.025
S5	38748	178	0.999131	207.7 ± 29.1	2.21 ± 0.35	0.253 ± 0.064
S6	38748	159	0.999148	189.7 ± 15.3	1.94 ± 0.41	0.291 ± 0.080
S7	38748	165	0.999209	196.0 ± 19.5	2.24 ± 0.13	0.235 ± 0.023
S8	38748	162	0.999045	199.7 ± 11.9	2.03 ± 0.05	0.268 ± 0.011
BLK	38748	216	0.999200	238.3 ± 5.5	3.38 ± 0.14	0.067 ± 0.011
FOS	38748	165	0.999028	216.3 ± 21.5	2.10 ± 0.16	0.237 ± 0.031
ORI	38748	210	0.999166	238.0 ± 12.3	3.12 ± 0.06	0.104 ± 0.007

## Data Availability

The data used to support the findings of this study are included within the article.
